# Endourologic Management of a 15-Year-Old Neglected, Fragmented, and Encrusted Ureteral Stent

**DOI:** 10.1089/cren.2018.0083

**Published:** 2018-12-17

**Authors:** Sarwar Noori Mahmood, Hewa Mahmoud Toffeq, Muhamed Hussen, Aram Karim, Choman Jamal, Aree Ali Said, Bryar Othman Aziz

**Affiliations:** ^1^ Department of Surgery, College of Medicine, University of Sulaymaniyah, Sulaymaniyah, Iraq.; ^2^ Department of Urology, Sulaymaniyah General Teaching Hospital, Sulaymaniyah, Iraq.

**Keywords:** Double-J stents, forgotten, broken, encrustations

## Abstract

***Background:*** Ureteral stents (Double-J stents) have been widely used in urology to prevent or relieve ureteral obstruction and have become an integral part of urologic practice. However, if ureteral stents are kept for a prolonged period or neglected, they can cause significant morbidity because of complications such as stent migration, encrustation, occlusion, stone formation, and fragmentation. Therefore, it is crucial to remove stents as soon as possible after they have served their purpose, to prevent complications and morbidity.

***Case Presentation:*** In this study, we present a case of a 28-year-old man who presented with broken extensively encrusted Double-J stent, with bulky stones at both ends of the stents that had been inserted 15 years ago, after an open right pyelolithotomy, and that was lost to follow-up. Cystolithotripsy, ureteroscopic laser lithotripsy, and two consecutive mini-percutaneous nephrolithotomies were necessary to extract the stent and the patient became stone free.

***Conclusion:*** Forgotten Double-J stents for long times with extensive encrustation and stone formation can be managed safely with a combined endourologic approach with minimal morbidity.

## Introduction and Background

The Double-J ureteral stent or pigtail stent has been an important tool in urologic practice, for managing ureteral obstructions because of intrinsic or extrinsic causes and for providing adequate internal drainage after ureteral surgeries or iatrogenic injuries and before any complex abdominal procedure for the identification and protection of the ureters.^1^ Nonetheless, their use can be accompanied by complications. Most complications occur after a prolonged indwelling of the stents. These complications include infection, stent migration, encrustation, stone formation, and stent fragmentation.^2^ When forgotten stents are encountered, treatment depends on the severity of the encrustation and stone formations at both ends of the stent. Stents have been surgically removed using various modalities ranging from extracorporeal shockwave lithotripsy (SWL), cystolitholapaxy, ureteroscopic laser lithotripsy, percutaneous nephrolithotomy (PCNL), and open surgery, either individually or in combination.^3^

In this study, we have described a single-session endourologic removal of a forgotten stent for 15 years that has been extensively encrusted, broken with stone formation.

## Case Presentation

A 28-year-old man presented with a history of right pyelolithotomy 15 years ago for a staghorn stone and an indwelling Double-J stent at 2002. The child and his parents did not follow up, despite being made aware of the Double-J stent removal. He presented 15 years later with a history of lower abdominal pain, dysuria, frequency, and intermittent hematuria for the past 3 months' duration.

For the past 3 years, the patient kept complaining of intermittent short episodes of burning micturition and lower abdominal pain, which were resolved with simple medications after visiting a local medical clinic in the rural area.

On examination, he was a healthy young man, his vital was stable, his abdomen was soft and not tender, there was a scar of a previous right pyelolithotomy, and all other systemic examinations were normal.

The complete blood count, renal function tests, and serum electrolytes were normal. The urine examinations revealed 10–15 pus cells with 20–30 RBCs, whereas the urine culture was negative of growth.

**FIG. 1.**
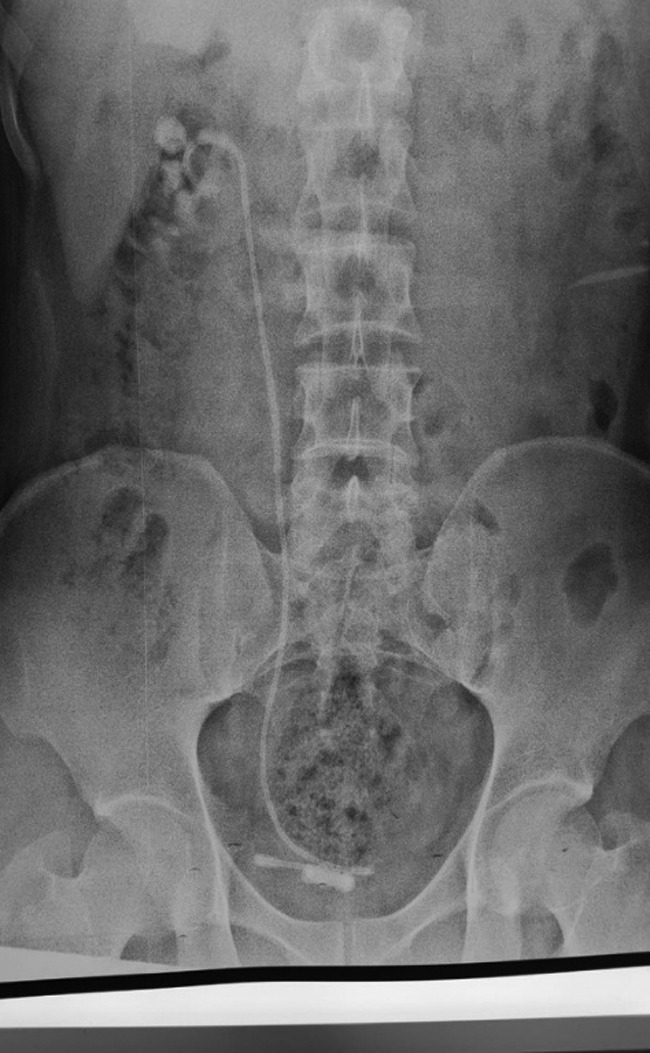
Plain KUB showed forgotten Double-J with the stone formation at both sides of the coils. KUB, kidney, ureter, and bladder radiograph.

**FIG. 2.**
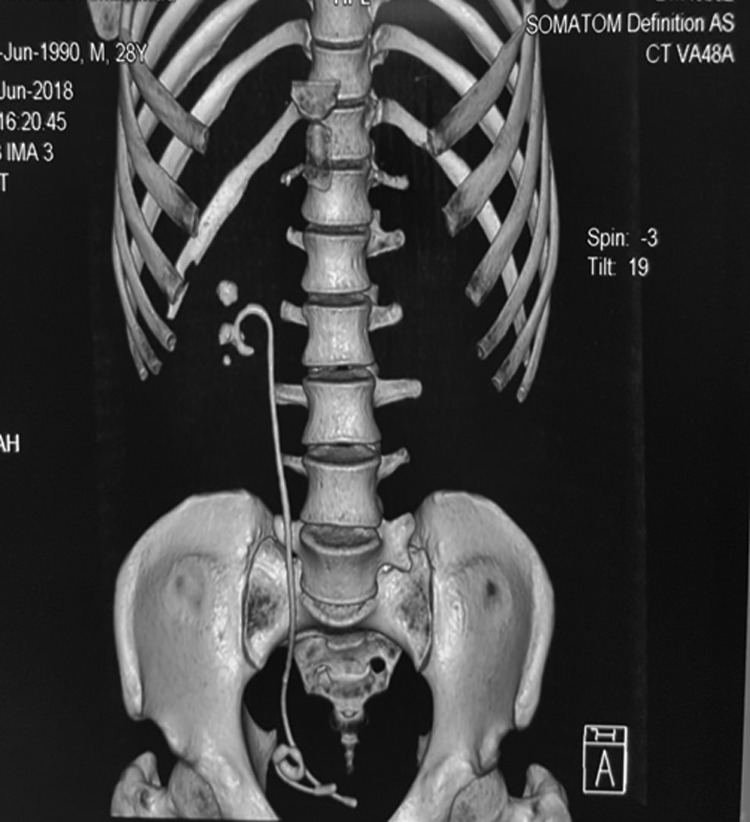
Native CT scan showed forgotten Double-J with a broken piece inside the urinary bladder.

A kidney, ureter, and bladder radiograph (KUB) (Fig. 1) and CT scan (Fig. 2) showed the broken distal coil of the Double-J stent inside the urinary bladder with a 2 × 2 cm vesical stone with an encrusted Double-J stent along the entire length, and multiple stones in the right kidney.

## Intervention

**FIG. 3.**
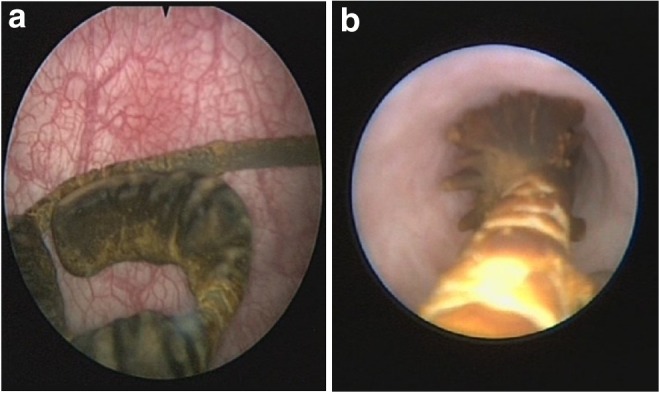
**(a)** Cystoscopic view showing the broken piece of the Double-J and encrustation; **(b)** ureteroscopic view of the Double-J showing extensive encrustation and stone formation.

The calculus along the broken distal coil of Double-J (Fig. 3a) was cleared with a lithoclast and distal coil (which had been broken and separated from the other part of the Double-J stent) removed alone. The ureteroscopy (URS) was done and stones with encrustations (Fig. 3b) along the Double-J were cleared using both the holmium laser (Calculase II; Karl Storz, Tuttlingen Germany) and lithoclast (Nidhi Meditech System, Ahmedabad, India) until the proximal coil inside the renal pelvis and the entire remaining part of the Double-J was removed (which was confirmed by a C-arm).

**FIG. 4.**
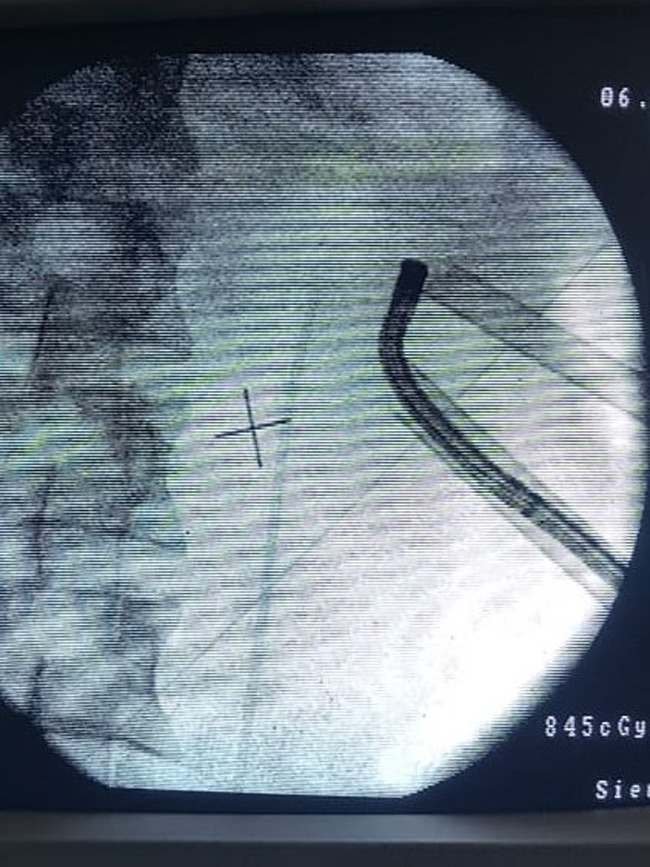
Two tract mini-PCNL and flexible nephroscope for clearance of the stones. PCNL, percutaneous nephrolithotomy.

**FIG. 5.**
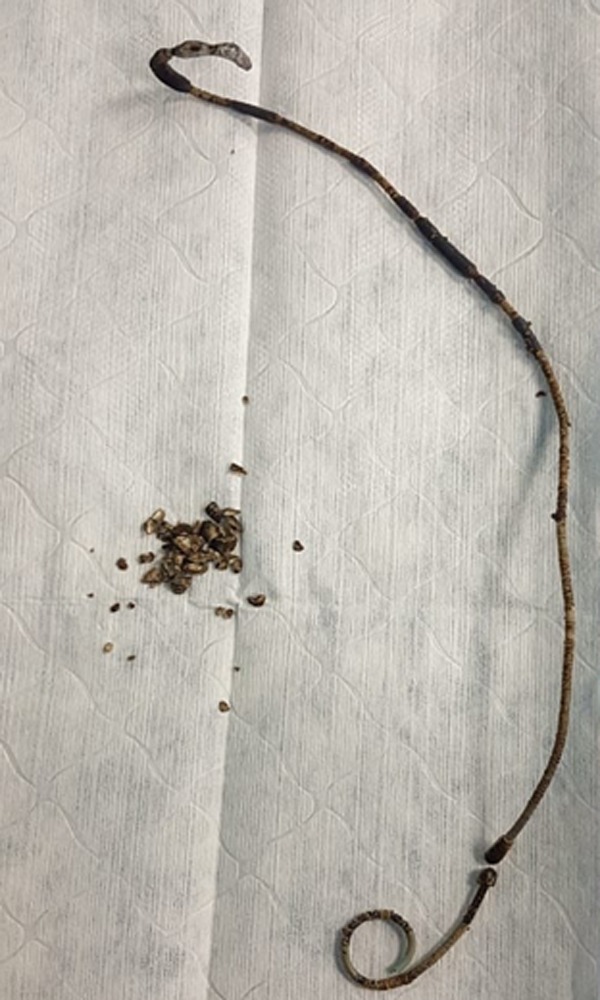
Removed Double-J stent showed a broken piece and the rest of Double-J with encrustation, with fragmented stones remove by PCNL.

**FIG. 6.**
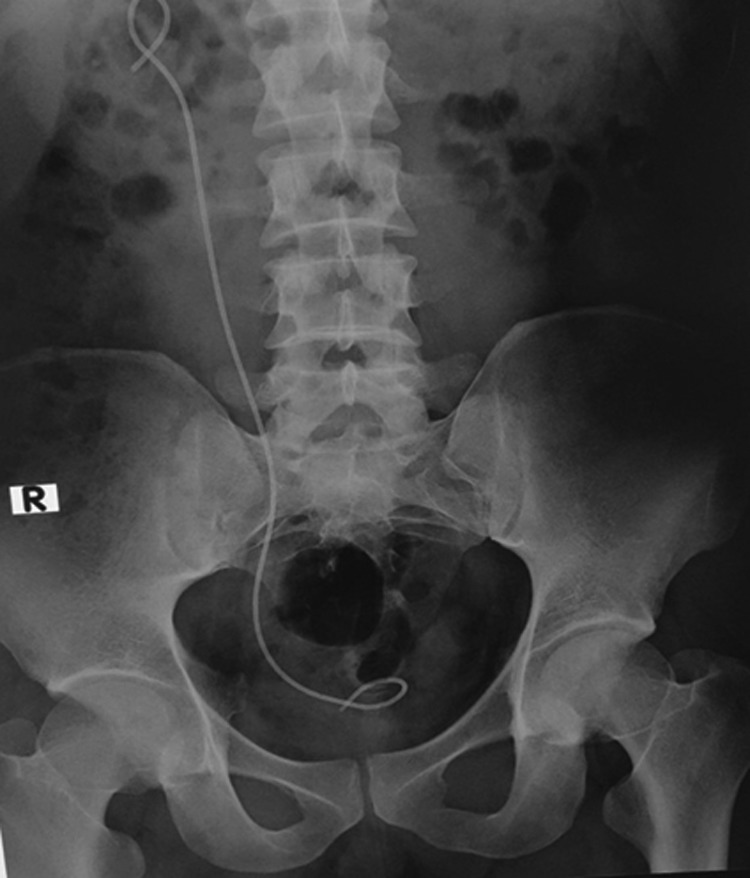
Postoperative KUB showed new inserted Double-J and stone-free status.

At the same session, the renal pelvis stone and the lower and mid calix stones were cleared by a two-tract mini-PCNL (20F Amplatz sheath) in a prone position; the intraoperative clearance was checked by a C-arm and flexible nephroscope (Fig. 4). Figure 5 shows the removed Double-J stent with a broken piece and the rest of the Double-J with encrustation, with fragments of stone removed by PCNL. Postoperative plain KUB demonstrated the new Double-J stent with no stone fragments (Fig. 6).

The patient was satisfied with the procedure and was free of symptoms after the removal of the new Double-J stent 10 days thereafter.

## Discussion

Ureteral stents (Double-J stents) have been widely used in urology practice to prevent or relieve the ureteral obstruction, usually for short determining periods between 2 and 12 weeks.^1^

However, forgotten or retained ureteral stents for a prolonged time could lead to numerous complications such as stent migration, stent occlusion, breakage, encrustation, and stone formation.^2^

The deposition of encrusted material on retained Double-J stents can occur in both infected and sterile urine. The rate of encrustation is dependent on the urinary composition, infection status, and metabolic or congenital abnormalities.^3^ A history of urolithiasis, infection, stent material, and the duration of stenting are regarded as important risk factors for encrustation.^3^

The exact reason for stent fragmentation is unclear and usually, stent fractures spontaneously occur after being *in situ* for a long time, because of hardening and the loss of tensile strength.^1^

SWL, ureteroscopic laser lithotripsy, PCNL, and open surgery, either alone or in combination, are employed for the management of a forgotten Double-J stent, depending on the location and severity of encrustation.^4^ Urologists well trained and sufficiently advanced in endourology can manage forgotten Double-J stents endoscopically, considering open surgery as a last resort when the endoscopic procedures fail.^4^

In this case, the calculus along the broken distal coil of Double-J was cleared with a lithoclast and the distal coil was removed alone. URS was conducted and the stones with encrustations along the Double-J were cleared using both the holmium laser and lithoclast until the proximal coil inside the renal pelvis and the Double-J was removed.

At the same session, the renal pelvis stone and the lower and mid calix stones were cleared by a two-tract mini-PCNL in a prone position; the intraoperative clearance was checked by a C-arm and flexible nephroscope.

The best treatment is the prevention of this complication. To evade the headache of forgotten stents, some surgeons advocate the use of a stent registry (name, address, and contact information) that tracks and recalls the clinicians about the stents that are long delayed for removal.^3,4^ In addition, the patient and their relatives should be adequately counseled about the presence of a foreign object, which should be removed after a specific interval, and the stents should not be left *in situ* for >3 months to minimize the risk of encrustations and complications.

## Conclusion

Double-J stents forgotten for a long time with extensive encrustation and stone formation can be managed safely with a combined endourologic approach with minimal morbidity.

## Abbreviations Used

CT: computed tomography
KUB: kidney, ureter, and bladder radiograph
PCNL: percutaneous nephrolithotomy
SWL: extracorporeal shock wave lithotripsy
URS: ureteroscopy

## References

[1] Ray RP, Mahapatra RS, Mondal PP, Pal DK Long-term complications of JJ stent and its management: A 5 years review. Urol Ann 2015;7:41–45 25657542 10.4103/0974-7796.148599 PMC4310115

[2] Nerli RB, Magdum PV, Sharma V, Guntaka AK, Hiremath MB, Ghagane S Forgotten/retained double J ureteric stents: A source of severe morbidity in children. Afr J Paediatr Surg 2016;13:32–35 27251521 10.4103/0189-6725.181704 PMC4955456

[3] Bidnur S, Huynh M, Hoag N, Chew B An indwelling ureteral stent forgotten for over 12 years. J Endourol Case Rep 2016;2:135–137 27579442 10.1089/cren.2016.0073 PMC4996618

[4] Bostanci Y, Ozden E, Atac F, Yakupoglu YK, Yilmaz AF, Sarikaya S Single session removal of forgotten encrusted ureteral stents: Combined endourological approach. Urol Res 2012;40:523–529 22160282 10.1007/s00240-011-0442-2

